# Notes

**Published:** 1898-04-09

**Authors:** 


### The Institutional Workshop.

### NOTES.

Mr. C. T. Ritchie, M.P., last week formally opened a block of six cottage homes erected by the Croydon Board of Guardians for the accommodation of pauper children belonging to the Union.

---

Lord Rothschild, the President of the Royal Hospital for Diseases of the Chest, City Road, has given 200 guineas to the festival dinner fund, and Messrs. N. M. de Rothschild have also contributed 100 guineas.

---

The ceremony of opening the new annexe of the Central London Ophthalmic Hospital, Gray's Inn Road, has been performed by the Lady Mayoress. The committee have in view the erection of a modern hospital upon the present site as soon as the funds will permit. The new building will now give the hospital 16 extra beds at a cost of £1,700.

---

The Baroness Burdett-Coutts, in making an appeal for the Poor Children's Dinner Society, states that the number of dinners given this winter amounts to 120,000. The prevalence of influenza and measles has caused additional strain on the funds of the society, and the continuance of cold weather makes the committee anxious to keep the dining-rooms open till after Easter.

---

A new wing of the Mansfield Accident Hospital has been opened by the Duchess of Portland. The Duke of Portland had officiated at a like function when the hospital was opened in 1890 as a Jubilee memorial. It then contained five beds, but for the past two years has been found too small for the demands of a growing colliery district, and this new ward, to contain ten extra beds, was thought a fitting commemoration of Her Majesty's record reign.

---

The Hackney Guardians at a recent meeting unanimously resolved "that the Board is of opinion that increased hospital accommodation for children is much needed in the districts of Hackney, Shoreditch, and Bethnal Green, and that therefore the completion of the North Eastern Hospital for Children, Hackney Road, which has been left unfinished since 1880, should, in the public interest, be no longer delayed."

---

The arrangement which has now come into force in London (except in the City) for the payment of jurymen at coroners' inquests will, we hope, help to remove what has been a very real grievance. The County Council have fixed the amount at 2s. per head for fifteen jurymen. It appears, however, that the fee will only go to those who are actually summoned, and will not be paid to those attending as substitutes, nor to persons summoned out of the street in cases of emergency. An occasional difficulty may then arise, but under ordinary circumstances the coroner's juryman will get some recompense for what is often a very disagreeable and irksome public duty—and we think he deserves it.

